# Blood Procalcitonin Level as a Diagnostic Marker of Pediatric Bacterial Meningitis: A Systematic Review and Meta-Analysis

**DOI:** 10.3390/diagnostics11050846

**Published:** 2021-05-08

**Authors:** Heeyeon Kim, Yun-Ho Roh, Seo-Hee Yoon

**Affiliations:** 1 Department of Pediatrics, Severance Children’s Hospital, Yonsei University College of Medicine, 50-1 Yonsei-ro, Seodaemun-gu, Seoul 03722, Korea; hykim0402@yuhs.ac; 2 Biostatistics Collaboration Unit, Department of Biomedical Systems Informatics, Yonsei University College of Medicine, 50-1 Yonsei-ro, Seodaemun-gu, Seoul 03722, Korea; yunhoroh@yuhs.ac

**Keywords:** bacterial meningitis, procalcitonin, children, diagnosis, meta-analysis, systematic review

## Abstract

Early diagnosis and treatment of bacterial meningitis in children are essential, due to the high mortality and morbidity rates. However, lumbar puncture is often difficult, and cerebrospinal fluid (CSF) culture takes time. This meta-analysis aims to determine the diagnostic accuracy of blood procalcitonin for detecting bacterial meningitis in children. We conducted a systematic search on electronic databases to identify relevant studies. Pooled sensitivity, specificity, and diagnostic odds ratio (DOR) were calculated, and a hierarchical summary receiver operating characteristic curve and area under the curve (AUC) were determined. Eighteen studies with 1462 children were included in the analysis. The pooled sensitivity, specificity, and the DOR of blood procalcitonin for detecting bacterial meningitis were 0.87 (95% confidence interval (CI): 0.78–0.93); 0.85 (95% CI: 0.75–0.91), and 35.85 (95% CI: 10.68–120.28), respectively. The AUC for blood procalcitonin was 0.921. Blood procalcitonin also showed higher diagnostic accuracy for detecting bacterial meningitis than other conventional biomarkers, including serum C-reactive protein and leukocyte count, CSF leukocyte and neutrophil count, and CSF protein and glucose levels. Blood procalcitonin can be a good supplemental biomarker with high diagnostic accuracy in detecting bacterial meningitis in children.

## 1. Introduction

Bacterial meningitis is an inflammation of the meninges associated with bacterial invasion [1,2]. The causative pathogens vary by age and geographic region, but *Streptococcus pneumoniae*, *Neisseria meningitidis*, *Haemophilus influenzae* type b (Hib), group B *Streptococcus*, and *Listeria monocytogenes* are the most common causes of bacterial meningitis. Introduction of the conjugate Hib vaccine (in 1990) and the heptavalent pneumococcal conjugate vaccine (in 2000), resulted in a decrease in the overall incidence of bacterial meningitis in countries where it has been implemented [3,4]. However, the mortality rate (up to 34% even if treated with antibiotics) and incidence of long-term sequelae (up to 50%) from bacterial meningitis among those affected have not changed and remain substantial [2,3]; thus, urgent diagnosis and prompt administration of appropriate antibiotics are crucial in patients with suspected bacterial meningitis.

Pediatric bacterial meningitis is diagnosed by the presence of clinical symptoms (i.e., fever, headache, lethargy, irritability, altered mental state, photophobia, nausea, vomiting, and stiff neck) and the examination of cerebrospinal fluid (CSF) obtained by lumbar puncture [3,5]. Specifically, identification of bacteria by culture or bacterial antigen detection (e.g., via a latex agglutination test) in the CSF can confirm a diagnosis. Typical CSF findings including elevated protein content (>100–150 mg/dl), a CSF: blood glucose ratio < 0.4–0.5, or decreased glucose (<40 mg/dl) with >80–90% neutrophils suggest the presence of bacterial meningitis [3,5,6].

Although CSF parameters (cell counts with differential protein and glucose levels) can help in the differential diagnosis of various types of meningitis, Gram staining and CSF culture are still traditionally the gold standard in confirming the diagnosis and identifying the bacterial pathogen [5]. However, false-negative results can occur when children receive antibiotic therapy before lumbar puncture. In addition, the likelihood of detecting bacteria on a CSF Gram stain depends on the pathogen and the number of organisms present, and CSF culture generally take time to produce a result [1,5].

In addition, lumbar punctures in children are difficult to perform and often cause bleeding (i.e., a traumatic lumbar puncture). Approximately 14–18% of attempted lumbar punctures are either traumatic or unsuccessful [7]. In neonates, incidence of traumatic/unsuccessful lumbar puncture is up to 46% [8]. Moreover, the results of CSF testing from traumatic lumbar puncture can be ambiguous and difficult to interpret [7]. Therefore, it is clinically important to find useful supplementary blood biomarkers to complement CSF examination for emergency diagnosis and timely antibiotic therapy in children with bacterial meningitis.

Thus far, there have been no exceptional biomarkers for diagnosing bacterial meningitis. Excluding existing CSF parameters (i.e., an increased percentage of polymorphonuclear leukocytes or decreased glucose concentration), CSF lactate has been suggested as a good single indicator for diagnosing bacterial meningitis [9,10,11]. However, for the aforementioned reasons, CSF lactate is not a suitable biomarker that can be easily tested in children. Conventional blood biomarkers, such as white blood cell (WBC) counts and C-reactive protein (CRP), have been widely used for diagnosing bacterial infections [12,13]. However, WBC counts and CRP levels can also be elevated in various types of systemic inflammations and viral infections [14], thus limiting their ability to distinguish bacterial from viral etiologies of meningitis [15,16].

Procalcitonin (PCT), one of the most validated markers of sepsis, is the precursor to the hormone calcitonin [17]. PCT is produced in the C cells of the thyroid gland and converted to calcitonin before it enters circulation under normal conditions [13,18,19]. In healthy individuals, PCT levels are typically under 0.10 ng/mL [12,20]. When a bacterial infection occurs, significant production of PCT by non-thyroidal tissues occurs throughout the body [18]. PCT levels have been shown to increase rapidly between 2–6 h and peak within 24 h following bacterial infection [18].

Previous systematic reviews have reported very high values for the sensitivity and specificity of blood PCT for bacterial meningitis in both children [21] and adults [22]. However, recent results of pediatric observational studies have not yet been pooled and reported; thus, our study aimed to provide an updated overview of the diagnostic accuracy of blood PCT for pediatric bacterial meningitis and compared it with that of the conventional blood and CSF biomarkers, which would determine whether blood PCT can replace the diagnostic role of blood or CSF parameters.

## 2. Materials and Methods

This systematic review was registered in the International Prospective Register of Systematic Reviews (PROSPERO; CRD42021186913) and conducted according to the Preferred Reporting Items for Systematic Reviews and Meta-Analysis (PRISMA) guidelines [23]. Two reviewers (S.H.Y. and H.K.) independently searched and selected the literature and performed data extraction and quality assessment. Any disagreements were resolved through discussion.

### 2.1. Search Strategy, Study Selection, and Eligibility Criteria

We searched PubMed, Embase, and the Cochrane Library for articles published until 30 March 2020. Search terms included “procalcitonin” and “meningitis,” and we restricted the database searches by the age filter (newborn to adolescent). Studies assessing the diagnostic accuracy of procalcitonin in serum or plasma for pediatric bacterial meningitis with sufficient data to construct a contingency table were included. Bacterial meningitis was defined as the presence of clinical symptoms of meningitis (i.e., fever, headache, neck stiffness, bulging fontanelle, or mental status changes) with detection of bacteria in the CSF by culture, Gram stain, or the bacterial antigen test [6]. Probable cases of bacterial meningitis were also included if patients had clinical symptoms of meningitis with CSF laboratory findings of leukocytosis >100 cells/mL with >80% neutrophils, CSF protein > 80 mg/dL, and glucose < 40 mg/dL; or CSF: blood glucose ratio < 0.4, with or without a positive blood culture [6,24,25].

We defined the pediatric age range as aged <18 years. No date restrictions were applied on the publication period. Bibliography of any eligible articles identified were also screened for additional relevant articles. Publications were excluded if they did not address bacterial meningitis or the accuracy of blood procalcitonin. We also excluded the following article types: reviews, letters, case reports, editorials, guidelines, and animal experiments. Repeated publications and non-English articles were also excluded.

### 2.2. Data Extraction

We retrieved the following data on each eligible study: first author, year of publication, location, age, sample size, sample type, cutoff value, diagnostic criteria, PCT assay methods, and diagnostic test results (true positive, false positive, false negative, or true negative). If studies were composed of multiple groups with different results, each group was considered as an individual study.

### 2.3. Quality Assessment

The Quality Assessment Tool for Diagnostic Accuracy Studies (QUADAS-2 score) was used to evaluate the methodological quality of the included studies [26]. It comprises four key domains: patient selection, index test, reference standard, and flow and timing. Each domain was judged as “low,” “high,” or “unclear” in terms of risk of bias, and the first three domains were also assessed in terms of concerns regarding applicability.

### 2.4. Statistical Analysis

Summary estimates of sensitivity, specificity, positive and negative likelihood ratios (LR+ and LR−), and diagnostic odds ratio (DOR) with their 95% confidence intervals (CI) were assessed using the bivariate model. Heterogeneity of sensitivity and specificity was assessed by a χ^2^ test (*p* < 0.10 indicated significant heterogeneity) and visually using a forest plot. The area under the curve (AUC) was obtained from a hierarchical summary receiver operating characteristic (HSROC) curve. The included studies used various cutoff values of procalcitonin to diagnose bacterial meningitis; thus, a threshold effect was anticipated. Therefore, we planned *a priori* subgroup analysis using a cutoff value of 0.5 ng/mL. The potential publication bias was assessed using Deeks’ funnel plot, where *p* < 0.1 indicated statistical significance. Data analysis was performed using the R package, version 4.0.3 (http://www.R-project.org, accessed on 11 December 2020) (R Foundation for Statistical Computing, Vienna, Austria), and Stata software, Version 16.1 (StataCorp, College Station, TX, USA).

## 3. Results

**Figure 1:**
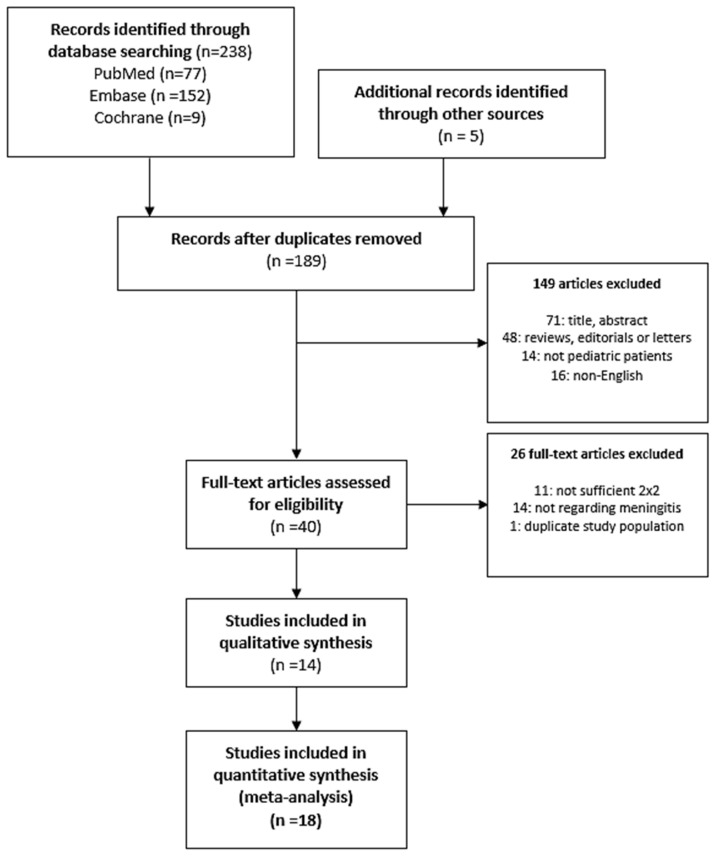
The flow diagram of the search and selection process.

The initial search identified a total of 243 titles and abstracts (77 from PubMed, 152 from Embase, 9 from Cochrane database, and 5 from other sources, such as reviewing references). Of these, after removing 54 duplicates, 189 articles were screened, and 149 articles were excluded based on eligibility criteria (Figure 1). From the 40 full-text reviews, 11 had insufficient data for 2 × 2 table construction, and in 14 studies, the disease of concern was not meningitis. One study was excluded due to a duplicate study population. The remaining 14 articles were eligible for data extraction, of which four articles reported two different sets of data using different cutoff values; thus, they were regarded as two separate studies. Finally, 18 studies comprising 1462 samples [10,16,27,28,29,30,31,32,33,34,35,36,37,38] were included (Figure 1).

### 3.1. Characterization of the Studies

The characteristics and diagnostic criteria of each study included in the meta-analysis are shown in the Table 1 and Table S1. The included studies were published between 1997 and 2019 and showed a wide geographical distribution (one from China; three, Egypt; four, France; two, Iran; one, Iraq; one, Nepal; one, Poland; three, Saudi Arabia; three, Spain; one, Switzerland; and two, Turkey). The age of enrolled patients ranged from newborns to 15.9 years (Table S1). Fourteen studies (77.7%) used serum PCT, and two studies (11.1%) used plasma PCT. The LUMItest PCT assay (Brahms Diagnostica, Berlin, Germany) was the most frequently studied PCT assay (*n* = 11, 61.1%). The included studies used varying cutoff values of PCT ranging from 0.05 ng/mL to 10 ng/mL, with a cutoff value of 0.5 ng/mL being the most commonly used (*n* = 7, 38.9%).

### 3.2. Quality Assessment of the Included Studies

**Figure 2:**
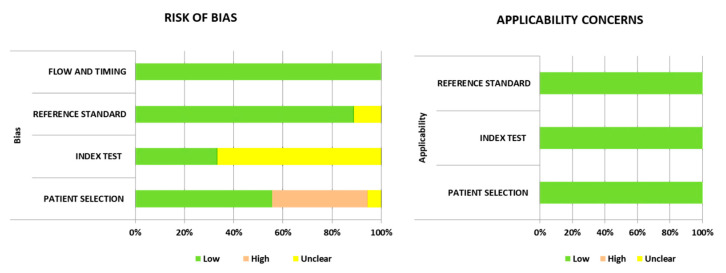
Summary of the risk of bias of the included studies (Quality assessment of the diagnostic accuracy studies-2, QUADAS-2).

Of the 18 included studies, seven (38.9%) studies had a high risk of bias in the patient selection domain because they did not exclude patients who had received previous antibiotic treatment. Twelve of 18 studies (66.7%) used optimal cutoff values, which maximized both sensitivity and specificity, instead of the predefined threshold and thus scored “unclear risk” in the index test domain. In the reference standard domain, we scored “low risk” on most studies (*n* = 16, 88.9%) because they used positive CSF culture with clinical symptoms and CSF laboratory findings as the reference standard. Two other studies used CSF laboratory findings (excluding CSF culture) with clinical symptoms as the reference standard; therefore, we scored them as “unclear risk.” All studies scored “low risk” in terms of bias in the flow and timing domain and “low concern” for applicability concerns (Figure 2). 

### 3.3. Pooled Diagnostic Accuracy of Procalcitonin

**Figure 3:**
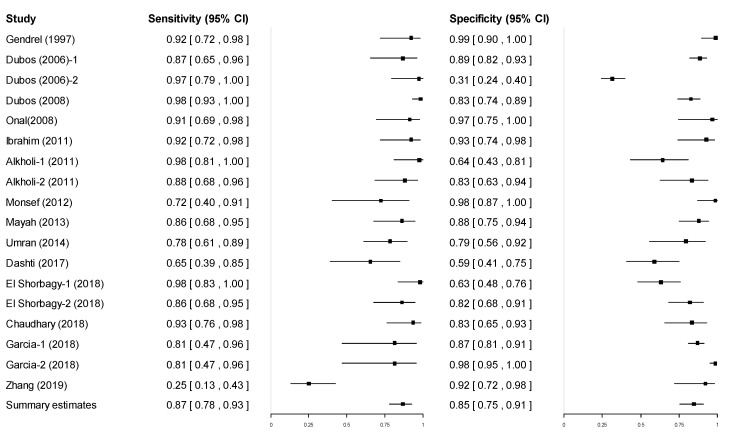
Coupled forest plots for sensitivity and specificity.

Forest plots of the sensitivity and specificity are shown in Figure 3. The summary estimate of sensitivity was 0.868 (95% CI: 0.777–0.925) and specificity was 0.845 (95% CI: 0.754–0.907). The summary estimates of LR+, LR−, and DOR were 5.600 (95% CI: 3.159–9.946), 0.156 (95% CI: 0.296–0.083), and 35.848 (95% CI: 10.680–120.283), respectively (Table S2). There was significant heterogeneity between studies in terms of sensitivity (χ^2^: 120.53; *p* < 0.001) and specificity (χ^2^: 291.57; *p* < 0.001).

**Figure 4:**
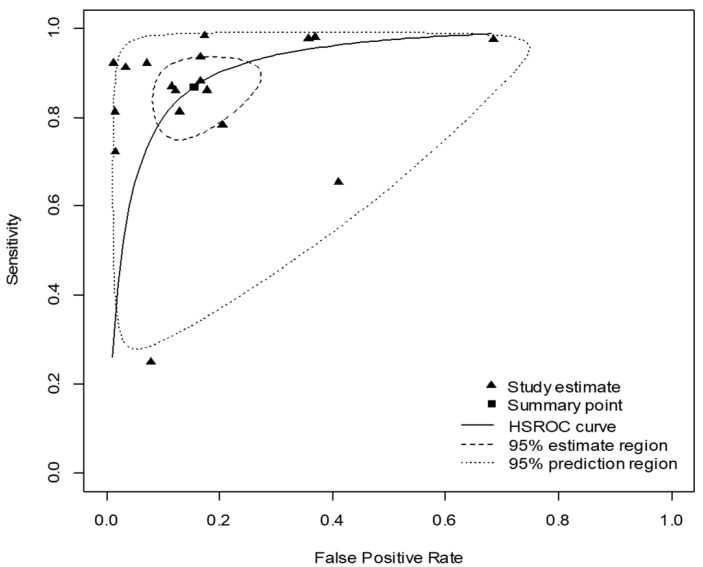
Hierarchical summary receiver operating characteristic (HSROC) curve of the diagnostic performance of procalcitonin for diagnosing pediatric bacterial meningitis. The area under the curve of the HSROC was 0.921.

**Figure 5:**
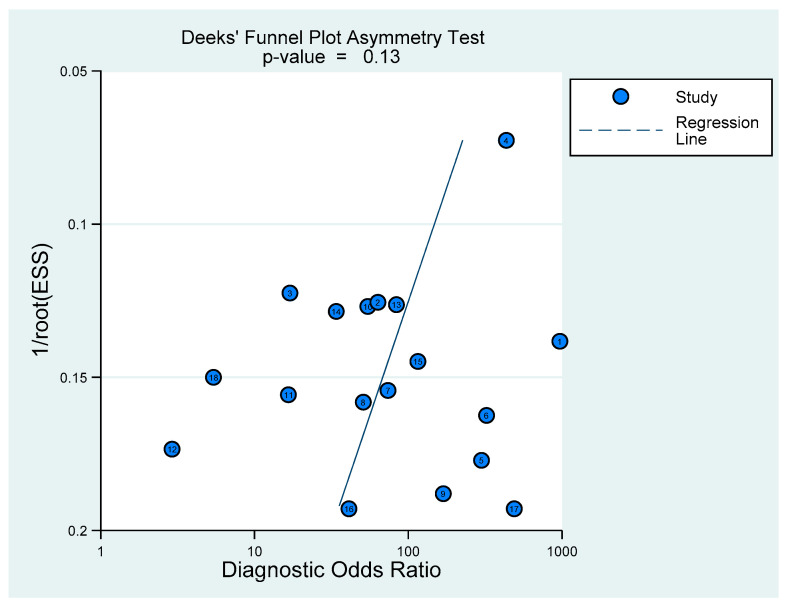
Deeks’ funnel plot for publication bias. ESS = effective sample size.

The area under the HSROC curve was 0.921 (Figure 4), which demonstrated that PCT had a high diagnostic accuracy for diagnosing pediatric bacterial meningitis. Deeks’ funnel plot revealed that there was no significant publication bias (*p* = 0.13) (Figure 5).

### 3.4. Subgroup Analysis according to the Cutoff Value

As the cutoff values differed among the included studies, we performed subgroup analysis, according to a pre-specified cutoff value, 0.5 ng/mL. Half of the included studies (*n* = 9) used cutoff values >0.5 ng/mL, and the other half used cutoff values ≤0.5 ng/mL. The subgroup with cutoff values ≤0.5 ng/mL had a higher pooled sensitivity (0.899 vs. 0.831) and similar specificity (0.844 vs. 0.851) to the subgroup with the cutoff values >0.5ng/mL. The subgroup with cutoff values ≤0.5 ng/mL also had a considerably higher pooled DOR (48.157 vs. 28.084) and diagnostic accuracy (AUC 0.935 vs. 0.908) (Table 2). Detailed accuracy estimates, HSROC curves, and heterogeneity test results, according to the subgroup, are provided in the Tables S3–S6 and Figures S1 and S2.

### 3.5. Comparison of Pooled Diagnostic Accuracy between Biomarkers

The pooled diagnostic accuracy for PCT compared with other conventional biomarkers, namely blood CRP level, CSF protein, and CSF glucose concentration, and blood WBC counts, CSF WBC counts, and CSF neutrophils were also performed. Among them, CSF protein concentration showed the highest sensitivity, and CSF neutrophils showed the highest specificity and DOR (Table 3). However, none of their pooled estimates of sensitivity, specificity, LR+, or the DOR were higher than that of blood PCT. Further detailed accuracy estimates and heterogeneity test results according to the specific biomarkers are provided in Tables S7–S18.

## 4. Discussion

Bacterial meningitis is an emergency medical condition. If untreated, the mortality rate is almost 100%, and neurological sequelae are common among survivors; thus, it requires rapid, accurate diagnosis with immediate initiation of empiric antibiotic treatment [1]. Our results showed that blood PCT is a highly accurate test for diagnosing pediatric bacterial meningitis. Specifically, the high pooled specificity and LR+ indicate that PCT is a good biomarker for ruling in bacterial meningitis in pediatric patients.

Compared with widely used infection biomarkers, CRP had a lower sensitivity, specificity, and LR+ than PCT. Furthermore, the pooled DOR for PCT, which is the best single indicator of diagnostic test performance [39], was almost three-times higher than that of CRP. Blood PCT has also several advantages over CRP. First, CRP starts to rise after 12–24 h and peaks at 48–72 h after the onset of infection, while PCT increases within 2–6 h and peaks within 6–24 h after the onset of infection [18,40]. Thus, PCT can be used as an earlier sensitive biomarker for the diagnosis of bacterial meningitis. PCT also has a significantly higher accuracy than CRP for discriminating between bacterial and viral infections or non-infective causes of inflammation [41]. In addition, unlike CRP, the PCT level is unaffected by the administration of nonsteroidal anti-inflammatory drugs (NSAIDs) or corticosteroids and by various inflammatory comorbidities (e.g., autoimmune diseases) [42,43,44,45]. These advantages are especially helpful for children who have previously been administered NSAIDs for symptomatic treatment prior to hospital visit [21].

Regarding the optimal diagnostic cutoff value, PCT showed increased power to diagnose bacterial meningitis in the subgroup analysis with cutoff values ≤0.5 ng/mL compared with the general analysis. Accordingly, we recommend the use of 0.5 ng/mL as the cutoff value of PCT for the detection of pediatric bacterial meningitis. However, further studies are required to determine the optimal cutoff values using a variety of PCT testing assays.

Another reason why PCT is a good auxiliary diagnostic test is that early clinical symptoms and signs are nonspecific and might be absent in the early phase among children [2]. Furthermore, bedside physical exam tests for neck stiffness, such as Brudzinski‘s sign and Kernig’s sign, are unable to distinguish between bacterial and viral meningitis, with sensitivity varying from 9 to 53% and specificity varying from 78 to 100% in children [46,47,48]. Consequently, to confirm the diagnosis of bacterial meningitis and identify the causative organism, CSF analysis obtained by lumbar puncture is necessary. However, the pooled estimates of sensitivity, specificity, and DOR were higher for blood PCT than those of all the measured CSF parameters.

In addition, failed or traumatic lumbar punctures can occur in up to 50% of lumbar punctures in pediatric patients and can cause diagnostic uncertainty, leading to unnecessary antibiotic treatment or prolonged hospitalization [49,50,51]. For this reason, measuring PCT can be helpful in situations when traumatic lumbar puncture or non-conclusive CSF findings occur in patients with suspected meningitis [21].

Despite these advantages, clinical application of PCT in diagnosing bacterial meningitis has a few limitations. PCT can be elevated in various bacterial infections, such as acute otitis media, pneumonia, and sepsis [52,53]. Thus, levels of blood PCT needs to be interpreted with caution in children with suspected acute bacterial meningitis along with other bacterial infections [54]. Blood PCT also has limited capacity to distinguish between acute febrile bacterial infections in the central nervous system (i.e., bacterial meningitis vs. brain abscess) [53]. Furthermore, PCT levels decrease with antibiotic use and may thus give misleading results in children who have recently been treated with antibiotics [55]. Finally, PCT is a more expensive diagnostic assay than CRP [56]. However, routine CRP testing can contribute to unnecessary hospital costs due to its low diagnostic value [57]. PCT-guided antibiotic therapy is associated with a reduction in antibiotic use and can help reduce overall healthcare costs [58,59,60]. The average turnaround time for CRP testing is about 50 min, which can delay the initiation of early treatment [61]. PCT assays, such as the Kryptor PCT assay [62] and LIAISON^®^ BRAHMS PCT^®^ II GEN [63], have a shorter turnaround time (about 20 min), which allows prompter initiation and appropriate use of antibiotics in children with bacterial meningitis.

A major strength of our review is that we searched multiple databases to ensure the current available evidence, which increased the sample size and thus provided more precise results than those of previous studies. Almost 1500 samples were included in our meta-analysis, which is almost double that of a previous review [21]. Second, most of the studies included had a prospective design, meaning that there was a lower risk of recall bias. Third, no significant publication bias was detected in the included studies.

There are several limitations in this study. First, significant heterogeneity was observed in the meta-analysis. The heterogeneity across studies may be due to the use of different reference standards, cutoff values, types of PCT assays, and different clinical conditions. We accepted the authors’ definitions of bacterial meningitis if they were based on the World Health Organization’s case definition criteria [6]. We also performed subgroup analysis according to the cutoff values, but could not perform subgroup analyses according to the other factors because of the limited information available. Second, we could not compare the pooled estimates of the diagnostic accuracy of CSF: blood glucose ratio and CSF PCT compared with blood PCT because there were too few studies that provided data with which these comparisons could have been performed. Previously, Onal et al. [29] reported that plasma PCT with a cutoff point of 0.5 ng/mL had similar sensitivity (0.937 vs. 0.93) and equal specificity (1.0) to CSF: blood glucose ratio (<0.6) for diagnosing pediatric bacterial meningitis. Zhang et al. [38] reported that CSF PCT with a cutoff point of 0.085 ng/mL showed a higher sensitivity (0.552 vs. 0.241) and a similar specificity (0.958 vs. 0.944) compared with serum PCT with a cutoff value of 5.91 ng/mL. However, Sanaei Dashti et al. [10] reported that CSF PCT with a 0.412 ng/mL cutoff showed a higher sensitivity (0.75 vs. 0.667) but a lower specificity (0.474 vs. 0.593) than serum PCT with a cutoff value of 0.6 ng/mL.

## 5. Conclusions

Current evidence suggests that blood PCT is a highly accurate diagnostic marker for pediatric bacterial meningitis and that it has higher diagnostic accuracy than blood CRP, WBC, and CSF parameters. Since the blood concentration of PCT rises within a few hours and peaks within 24 h, blood PCT can help differentiate between viral and bacterial etiology early in children with suspected meningitis. In addition, PCT can be used for monitoring response to antimicrobial therapy. Therefore, blood PCT help to reduce unnecessary prescription and duration of antibiotic therapy. In conclusion, blood PCT can be a useful diagnostic biomarker for bacterial meningitis in children. Future studies are needed to observe whether blood PCT can serve as a standalone biomarker for the diagnosis of pediatric bacterial meningitis in various clinical settings.

## Figures and Tables

**Table 1:** Characteristics of the included studies.

First Author (Year), Reference	Country	BM (*n*)	Non-BM (*n*)	Sample Type	Cutoff (ng/mL)	PCT Assay	Time of PCT Assessment
Gendrel (1997) [27]	France	18	41	Plasma	5	LUMItest PCT (Brahms Diagnostica, Berlin, Germany)	On admission
Dubos-1 (2006) [28]	France	18	134	Serum	0.5	LUMItest PCT (Brahms Diagnostica, Berlin, Germany)	On admission
Dubos-2 (2006) [28]	France	18	134	Serum	0.2	LUMItest PCT (Brahms Diagnostica, Berlin, Germany)	On admission
Dubos (2008) [16]	Switzerland, France, Spain, Turkey, Poland	90	100	Serum	0.5	Lumitest PCT (Brahms Diagnostica, Berlin, Germany)	On admission
Onal (2008) [29]	Turkey	16	14	Plasma	0.5	Lumitest PCT (Brahms Diagnostica, Berlin, Germany)	On admission
Ibrahim (2011) [30]	KSA	18	20	Serum	0.5	Immunoluminometric assay (Brahms Diagnostica, Berlin, Germany)	On admission
Alkholi-1 (2011) [31]	Egypt	20	20	Serum	2	Lumitest PCT (Brahms Diagnostica, Berlin, Germany)	On diagnosis
Alkholi-2 (2011) [31]	Egypt	20	20	Serum	10	Lumitest PCT (Brahms Diagnostica, Berlin, Germany)	On diagnosis
Monsef (2012) [32]	Iran	8	32	Serum	0.5	Lumitest PCT (Brahms Diagnostica, Berlin, Germany)	Before antibiotic therapy
Mayah (2013) [33]	Egypt	24	44	Serum	3.3	Lumitest PCT (Brahms Diagnostica, Berlin, Germany)	On admission
Umran (2014) [34]	Iraq	29	16	Serum	0.05	ELIZA M6 (NA, USA)	On admission
Sanaei Dashti (2017) [10]	Iran	12	27	Serum	0.6	Human PCT ELISA (BT Laboratory, Shanghai, China)	On admission
El Shorbagy-1 (2018) [35]	KSA	24	41	Serum	2	Lumitest PCT (Brahms Diagnostica, Berlin, Germany)	On diagnosis
El Shorbagy-2 (2018) [35]	KSA	24	41	Serum	10	Lumitest PCT (Brahms Diagnostica, Berlin, Germany)	On diagnosis
Chaudhary (2018) [36]	Nepal	22	26	Serum	0.5	Maglumi PCT (Snibe Diagnostics, Shenzhen, China)	On admission
Garcia-1 (2018) [37]	Spain	7	165	NA	0.5	NA	At the time of ED visit
Garcia-2 (2018) [37]	Spain	7	165	NA	2	NA	At the time of ED visit
Zhang (2019) [38]	China	29	18	Serum	5.91	VIDAS BRAHMS PCT (Biomerieux, Marcy l’Etoile, France)	On admission

BM, bacterial meningitis; ED, emergency department; KSA, Kingdom of Saudi Arabia; NA, not available; PCT, procalcitonin.

**Table 2:** Summary estimates of the diagnostic accuracy of procalcitonin for diagnosis of bacterial meningitis according to the cutoff value.

Cutoff	Number of Studies	Sensitivity (95% CI)	Specificity (95% CI)	LR+ (95% CI)	LR− (95% CI)	DOR (95% CI)	AUC
≤0.5 pg/mL	9	0.899 (0.81–0.949)	0.844 (0.702–0.96)	5.763 (2.718–23.725)	0.12 (0.271–0.053)	48.157 (10.043–446.588)	0.935
>0.5 pg/mL	9	0.831 (0.647–0.93)	0.851 (0.706–0.931)	5.577 (2.201–13.478)	0.199 (0.5–0.075)	28.084 (4.401–179.261)	0.908

AUC, area under the curve; CI, confidence interval; DOR, diagnostic odds ratio; LR+, positive likelihood ratio; LR−, negative likelihood ratio.

**Table 3:** Summary estimates of the diagnostic accuracy for other biomarkers.

Biomarkers	Number of Studies	Sensitivity (95% CI)	Specificity (95% CI)	LR+ (95% CI)	LR− (95% CI)	DOR (95% CI)
CRP	10	0.797 (0.741–0.844)	0.725 (0.665–0.777)	2.894 (2.213–3.781)	0.28 (0.39–0.201)	10.334 (5.679–18.808)
WBCs	5	0.659 (0.504–0.786)	0.713 (0.587–0.813)	2.294 (1.22–4.195)	0.479 (0.845–0.263)	4.794 (1.443–15.925)
CSF WBCs	4	0.733 (0.601–0.834)	0.669 (0.58–0.748)	2.217 (1.43–3.309)	0.399 (0.689–0.223)	5.556 (2.076–14.87)
CSF neutrophils	4	0.793 (0.377–0.96)	0.749 (0.519–0.892)	3.158 (0.784–8.895)	0.277 (1.201–0.045)	11.403 (0.652–199.271)
CSF protein	4	0.838 (0.699–0.92)	0.658 (0.55–0.753)	2.452 (1.552–3.718)	0.247 (0.548–0.107)	9.934 (2.833–34.833)
CSF glucose	3	0.563 (0.172–0.889)	0.193 (0.145–0.253)	0.698 (0.201–1.189)	2.264 (5.718–0.44)	0.308 (0.035–2.701)

AUC, area under the curve; CI, confidence interval; CRP, C-reactive protein; CSF, cerebrospinal fluid; DOR, diagnostic odds ratio; LR+, positive likelihood ratio; LR−, negative likelihood ratio; WBC, white blood cell.

## Data Availability

Available in this manuscript.

## Supplementary Materials

The following are available online at https://www.mdpi.com/article/10.3390/diagnostics11050846/s1, Figure S1: Hierarchical summary receiver operating characteristic (HSROC) curve in studies with a cut-off value ≤ 0.5 ng/mL, Figure S2: Hierarchical summary receiver operating characteristic (HSROC) curve in studies with a cut-off value > 0.5 ng/mL, Table S1: Characteristics of included studies, Table S2: Summary estimates of the overall diagnostic accuracy of blood procalcitonin, Table S3: Summary estimates of the diagnostic accuracy of blood procalcitonin in studies with a cut-off value ≤ 0.5 ng/mL, Table S4: Heterogeneity among studies with a cut-off value ≤ 0.5 ng/mL, Table S5: Summary estimates of the diagnostic accuracy of blood procalcitonin in studies with a cut-off value > 0.5 ng/mL, Table S6: Heterogeneity among studies with a cut-off value > 0.5 ng/mL, Table S7: Diagnostic performance of C-reactive protein, Table S8: Heterogeneity among studies evaluating C-reactive protein level, Table S9: Diagnostic performance of white blood cell count, Table S10: Heterogeneity among studies evaluating white blood cell count, Table S11: Diagnostic performance of white blood cell count in the cerebrospinal fluid, Table S12: Heterogeneity among studies evaluating white blood cell count in the cerebrospinal fluid, Table S13: Diagnostic performance of neutrophils in the cerebrospinal fluid, Table S14: Heterogeneity among studies evaluating neutrophils in the cerebrospinal fluid, Table S15: Diagnostic performance of protein level in the cerebrospinal fluid, Table S16: Heterogeneity among studies evaluating protein level in the cerebrospinal fluid, Table S17: Diagnostic performance of glucose level in the cerebrospinal fluid, Table S18: Heterogeneity among studies evaluating glucose level in the cerebrospinal fluid.
