# Supplementary material for: Blood Procalcitonin Level as a Diagnostic Marker of Pediatric Bacterial Meningitis: A Systematic Review and Meta-Analysis
Source: Diagnostics (Basel). 2021 May 8;11(5):846. doi: 10.3390/diagnostics11050846 (PMC8151301; doi:10.3390/diagnostics11050846)
Supplement: Supplementary file 1 [file diagnostics-11-00846-s001.zip › diagnostics-1190689-supplementary.pdf]

# Blood procalcitonin level as a diagnostic marker of pediatric bacterial meningitis: a systematic review and meta-analysis

## Supplementary Materials

**Table S1.** Characteristics of included studies.

| Authors | Study period | BM | Non-BM | Age, mean (range) | Definition of BM | Definition of non-BM | Etiologies (BM) | Etiologies (non-BM) |
|---|---|---|---|---|---|---|---|---|
| **Gendrel (1997)** | Jan. 1994–Dec. 1995 | BM | VM | NA | Positive bacterial culture of CSF | Positive symptoms of meningitis, CSF leukocytes counts of > 20/μL, negative bacterial cultures, and bacterial antigen detection in CSF | *Neisseria meningitidis* (n=8), *Haemophilus influenzae* type b (n = 5), *Streptococcus pneumoniae* (n = 3), *Escherichia coli* (n = 1), and *Listeria monocytogenes* (n = 1) | Viral culture: Enterovirus (n = 4), Adenovirus (n = 2), Varicella-zoster virus (n = 1)<br><br>RT-PCR: Enterovirus (n = 17) |
| **Dubos-1 (2006)** | BM (Jan 1995 – Oct. 2004), AM (Jan. 2000 – Apr. 2004) | BM | AM | 4.6 yrs* (0.2 – 14.9 yrs) | Acute onset of meningitis ( ≥7 WBC/ $mm^3$ in CSF) with documented bacterial infection in the CSF (direct examination, culture, or latex agglutination) or blood culture. | Acute onset of meningitis ( ≥7 WBC/ $mm^3$ in CSF) and the absence of any bacterial meningitis criteria | *Streptococcus pneumoniae* (n = 10), *Neisseria meningitidis* (n = 9), *Haemophilus influenzae* type b (n = 1), and Group B *Streptococcus* (n = 1) | NA |
| **Dubos-2 (2006)** | BM (Jan. 1995 – Oct. | BM | AM | 4.6 yrs* (0.2 – 14.9 yrs) | Acute onset of meningitis ( ≥7 WBC/ $mm^3$ in CSF) with | Acute onset of meningitis ( ≥7 WBC/ $mm^3$ in CSF) and the | *Streptococcus pneumoniae* (n = 10), *Neisseria meningitidis* | NA |

| | | | | | | | | |
|---|---|---|---|---|---|---|---|---|
| | 2004), AM (Jan. 2000 – Apr. 2004) | | | | documented bacterial infection in the CSF (direct examination, culture, or latex agglutination) or blood culture. | absence of any bacterial meningitis criteria | (n = 9), *Haemophilus influenzae* type b (n = 1), and Group B *Streptococcus* (n = 1) | |
| **Dubos (2008)** | 1996 – 2005 | BM | AM | 4.8 yrs (0.1 – 15.9 yrs) | Acute onset of meningitis ( ≥7 WBC/ $mm^3$ in CSF) with documented bacterial infection in the CSF (direct examination, culture, latex agglutination, or PCR) or blood culture. | Acute onset of meningitis ( ≥7 WBC/ $mm^3$ in CSF) and the absence of any bacterial meningitis criteria | *Neisseria meningitidis* (n = 45), *Streptococcus pneumoniae* (n = 32), *Haemophilus influenzae* (n = 7), and *Streptococcus agalactiae* (n = 4) | NA |
| **Onal (2008)** | Jan. 2001 – Jun. 2001 | BM | VM | BM: 3.2yrs (3 mo–12 yrs)<br><br>VM: 5.2 yrs (6 mo – 13 yrs) | Documentation of infection in CSF culture, CSF pleocytosis (>10 cells/$mm^3$) and predominance of polymorphonuclear neutrophilic leukocyte in direct examination of CSF, presence of bacteria in direct examination of CSF, CSF glucose: blood glucose ratio < 0.6, increase in CSF protein, and positivity of bacterial antigen in CSF | Patient with predominance of lymphocytes in CSF pleocytosis, slight increase in protein, and with normal glucose levels in CSF, no bacteria in direct examination and negativity of bacteria antigens of CSF | NA | NA |
| **Ibrahim (2011)** | Mar. 2005 – Feb. 2008 | BM | Non-BM | NA (2 mo – 10 yrs) | If the CSF laboratory findings showed: (increased protein >2 g/L, decreased glucose ratio < 0.4, leukocyte count > 1500 × $10^6$/L and polymorph nuclear leukocyte domination), identification of bacterial | If no bacteria were documented on Gram-stain or bacterial culture of CSF, lymphocyte predominance of CSF cells, reduced protein level, and increased glucose ratio > 0.5 | *Neisseria meningitidis* (n = 5), *Streptococcus pneumoniae* (n = 5), *Haemophilus influenzae* type b (n = 4), *Escherichia coli* (n = 2), and *Staphylcoccus aureus* (n = 2) | NA |

| | | | | | | | | |
|---|---|---|---|---|---|---|---|---|
| | | | | | agents in gram staining and/or positive bacterial culture of CSF | | | |
| **Alkholi-1 (2011)** | Jul. 2007 – Dec. 2007 | BM | VM | 5 yrs (4 mo – 12 yrs) | If the CSF laboratory findings showed: (increased protein ≥ 2 g/L, decreased glucose ratio ≤ 0.4, and leukocyte count ≥ 1500 × $10^6$/L and polymorphonuclear leukocyte domination), identification of bacterial agents in Gram staining, and/or positive bacterial culture of CSF | If the viral culture, serological testing, pleocytosis, or RT-PCR of CSF were positive, and the bacterial culture of CSF was negative. | *Neisseria meningitidis* (n = 6), *Streptococcus pneumoniae* (n = 6), *Haemophilus influenzae* (n = 4), *Escherichia coli* (n = 2), and *Pseudomonas aeruginosa* (n = 1) | CSF serology: Mumps virus (n = 3), Measles virus (n = 2), Varicella-zoster virus (n = 2)<br><br>RT-PCR: Enterovirus (n = 12) |
| **Alkholi-2 (2011)** | Jul. 2007 – Dec. 2007 | BM | VM | 5 yrs (4 mo – 12 yrs) | If the CSF laboratory findings showed: (increased protein ≥2 g/L, decreased glucose ratio ≤ 0.4, and leukocyte count ≥ 1500 × $10^6$/L and polymorphonuclear leukocyte domination), identification of bacterial agents in Gram staining, and/or positive bacterial culture of CSF | If the viral culture, serological testing, pleocytosis, or RT-PCR of CSF were positive, and the bacterial culture of CSF was negative. | *Neisseria meningitidis* (n = 6), *Streptococcus pneumoniae* (n = 6), *Haemophilus influenzae* (n = 4), *Escherichia coli* (n = 2), and *Pseudomonas aeruginosa* (n = 1) | CSF serology: Mumps virus (n = 3), Measles virus (n = 2), Varicella-zoster virus (n = 2)<br><br>RT-PCR: Enterovirus (n = 12) |
| **Monsef (2012)** | Apr. 2009 – Sep. 2010 | BM | Healthy | newborns | Clinical (hypothermia or fever, lethargy, convulsion, poor feeding, abdominal distension, organomegaly, apnea, tachypnea, cyanosis, respiratory distress, brady- or tachycardia) and laboratory findings [leukocytosis (WBC >2 5,000/$mm^3$), leukopenia (WBC < 5,000/$mm^3$), | Healthy newborns with indirect, non hemolytic jaundice without clinical and laboratory findings in favor of infection, who were treated with phototherapy. | NA | NA |

| | | | | | | | | |
|---|---|---|---|---|---|---|---|---|
| | | | | | thrombocytopenia (PLT < 150,000/mm³) and positive quantitative CRP] in favor of bacterial infection (before antibiotic therapy) and a positive CSF bacterial culture | | | |
| **Mayah (2013)** | NA | BM | AM + Control group | 69.3 mo (3 – 156 mo) | Children presenting with clinical symptoms of meningitis; fever, headache, stiff neck, bulging fontanelle, or mental status changes, CSF with an elevated protein (> 100 mg/dl), decreased glucose (<40 mg/dl) or leukocytosis (WBC > 100/mm³) with at least 80% neutrophils. Identification of bacteria directly by Gram stain smears or cultures from blood or CSF or indirectly by latex agglutination test of CSF | AM: presence of acute onset of meningitis symptoms, WBC of > 5/mm³ of which > 50% were mononuclear/lymphocyte cells with the absence of any bacterial meningitis.<br><br>Control group: absence of inflammatory cells in CSF (WBC < 5/mm³) and sterile bacteriologic findings in afebrile children with positive meningeal signs. | *Neisseria meningitidis* (n = 12), *Haemophilus influenzae* type b (n = 8), and *Streptococcus pneumoniae* (n = 6) | NA |
| **Umran (2014)** | Apr. 2013 – Oct. 2013 | BM | Non-BM | 17.7 mo (1 – 60 mo) | Positive clinical history and physical examination (fever, abnormal movement, irritability, bulging fontanel, meningeal irritation signs) and findings of CSF (increased protein > 0.2 g/L, decreased glucose ratio < 0.4, leukocyte count > 1500×10⁶/L and polymorphonuclear leukocyte domination) | Patients with clinical suspicious of acute meningitis and with none of the BM findings. | NA | NA |

| | | | | | | | | |
|---|---|---|---|---|---|---|---|---|
| **Sanaei Dashti (2017)** | Oct. 2012 – Nov. 2013 | BM | VM/AM | 42.7 mo (21 d – 144 mo) | a) Definite BM: samples with positive Gram staining, culture, or PCR (for *Streptococcus pneumoniae, Neisseria meningitidis*, *Haemophilus influenzae, Listeria monocytogenes,* and Group B *streptococcus*)<br><br>b) Presumed BM: The presence of clinical picture of meningitis with at least 2 of the following: protein ≥ 80, glucose ≤ 40, WBC ≥ 300, and/or polymorphnuclear cell predominancy. | a) Definite VM/AM: samples with positive PCR of Enterovirus, Human Herpesvirus, Varicella zoster virus, and Epstein-Barr virus.<br><br>b) Presumed VM/AM: Presence of clinical symptoms of meningitis with any CSF lacking the bacterial characteristics (previously mentioned in the presumed BM group) | *Streptococcus pneumoniae* (n = 5), *Haemophilus influenzae* (n = 4), and *Staphylcoccus aureus* (n = 1) | Herpes simplex virus type 1 (n = 1), Enterovirus (n = 1) |
| **El Shorbagy -1 (2018)** | Jun. 2015 – Dec. 2016 | BM | AM + Control group | BM: 7.6 yrs (4 mo – 14 yrs)<br><br>AM: 5.8 yrs (4 mo – 12 yrs) | Increased protein > 2 g/L, decreased glucose ratio < 0.4, leukocyte count > 1500x $10^6$/L and predominance of polymorph nuclear leukocyte in CSF. Identification of bacterial agents in Gram stain and/or bacterial culture | No bacteria on Gram stain or bacterial culture of CSF, reduced protein level, increased glucose ratio > 0.5 and predominance of lymphocyte in CSF. Identification of viral agents in viral culture and/or PCR | *Neisseria meningitidis* (n = 8), *Streptococcus pneumoniae* (n = 6), *Haemophilus influenzae* (n = 5), *Escherichia coli* (n = 2), *Staphylcoccus aureus* (n = 1), and *Pseudomonas aeruginosa* (n = 1) | NA |
| **El Shorbagy -2 (2018)** | Jun. 2015 – Dec. 2016 | BM | AM + Control group | BM: 7.6 yrs (4 mo – 14 yrs)<br><br>AM: 5.8 yrs (4 mo – 12 yrs) | Increased protein > 2 g/L, decreased glucose ratio < 0.4, leukocyte count > 1500x $10^6$/L and predominance of polymorph nuclear leukocyte in CSF. Identification of bacterial agents in Gram stain and/or bacterial culture | No bacteria on Gram stain or bacterial culture of CSF, reduced protein level, increased glucose ratio > 0.5 and predominance of lymphocyte in CSF. Identification of viral agents in viral culture and/or PCR | *Neisseria meningitidis* (n = 8), *Streptococcus pneumoniae* (n = 6), *Haemophilus influenzae* (n = 5), *Escherichia coli* (n = 2), *Staphylcoccus aureus* (n = 1), and *Pseudomonas aeruginosa* (n = 1) | NA |

| | | | | | | | | |
|---|---|---|---|---|---|---|---|---|
| **Chaudhary (2018)** | Aug. 2016 – Dec. 2016 | BM | Non-BM | 24 mo* (3 mo – 15 yrs) | If CSF showed bacteria in Gram staining and/or culture, predominantly neutrophilic cells, glucose levels less than two-third of blood glucose or elevated CSF protein (> 45 mg/dL) despite receiving antibiotics for 1–3 days | If CSF showed no growth of organisms, had predominantly lymphocytic counts, and normal (15–45 mg/dL) or slightly increased (up to 60 mg/dL) protein. | *Staphylococcus aureus* (n = 3), Acinetobacter sp. (n = 3), *Streptococcus pneumoniae* (n = 2), Enterococcus sp. (n = 2), and *Escherichia coli* (n = 1) | NA |
| **Garcia-1 (2018)** | Oct. 2012 – Sep. 2015 | BM | AM (VM+ Non-specific meningitis) | NA (2 – 14 yrs) | Detection of a bacterial pathogen in the CSF (positive bacterial culture and/or positive Gram stain and/or bacterial genomic detection) or in the blood culture with associated pleocytosis | VM: positive enteroviral culture or positive enteroviral PCR in CSF<br><br>Non-specific meningitis: pleocytosis and no detection of a bacterial pathogen or enterovirus in CSF and blood. | *Neisseria meningitidis* (n = 4), *Streptococcus pneumoniae* (n = 4), and *Haemophilus influenzae* (n = 1) | Enterovirus (n = 134) |
| **Garcia-2 (2018)** | Oct. 2012 – Sep. 2015 | BM | AM (VM+ Non-specific meningitis) | NA (2 – 14 yrs) | Detection of a bacterial pathogen in the CSF (positive bacterial culture and/or positive Gram stain and/or bacterial genomic detection) or in the blood culture with associated pleocytosis | VM: positive enteroviral culture or positive enteroviral PCR in CSF<br><br>Non-specific meningitis: pleocytosis and no detection of a bacterial pathogen or enterovirus in CSF and blood. | *Neisseria meningitidis* (n = 4), *Streptococcus pneumoniae* (n = 4), and *Haemophilus influenzae* (n = 1) | Enterovirus (n = 134) |
| **Zhang (2019)** | Jan. 2014 – Dec. 2016 | BM | VM | BM: 5.2 yrs (NA)<br><br>VM: 5.4 yrs (NA) | If the CSF showed increased protein > 100 mg/dL or decreased glucose < 40 mg/dL or leukocyte count >100 WBC/mm$^3$ with at least 80% neutrophils, identification of bacterial agents in gram staining, and/or positive bacterial culture. | Those with a CSF pleocytosis of with lymphocyte predominance or protein levels of 50 to 200 mg/dL, and normal glucose levels with a negative bacterial culture and Gram stain | *Streptococcus pneumoniae* (n = 5), *Streptococcus agalactiae* (n = 2), *Listeria monocytogenes* (n = 2), *Enterococcus faecium* (n = 1), *Staphylococcus aureus* (n = 1), *Escherichia coli* (n = 1), *Haemophilus influenzae* | Epstein-Barr virus (n = 1), Varicella-zoster virus (n = 2) |

| | | | | | | | | |
|---|---|---|---|---|---|---|---|---|
| | | | | | | | (n = 1), and *Clostridium perfringens* (n = 1) | |

* Median age. AM, aseptic meningitis; ANC, absolute neutrophil count; BM, bacterial meningitis; CRP, C-reactive protein; CSF, cerebrospinal fluid; NA, not available; non-BM, non-bacterial meningitis; PCR, polymerase chain reaction; PLT, platelet; RT-PCR, reverse transcriptase polymerase chain reaction; VM, viral meningitis; WBC, white blood cell.

**Table S2.** Summary estimates of the overall diagnostic accuracy of blood procalcitonin.

| Study | Sensitivity | Specificity | +LR | -LR | DOR |
|---|---|---|---|---|---|
| Gendrel (1997) | 0.921 (0.719 ,0.982) | 0.988 (0.896 ,0.999) | 77.368 (4.905 ,1220.458) | 0.080 (0.017 ,0.371) | 968.333 (37.584 ,24948.556) |
| Dubos (2006)-1 | 0.868 (0.655 ,0.958) | 0.885 (0.820 ,0.929) | 7.564 (4.588 ,12.471) | 0.149 (0.047 ,0.473) | 50.884 (12.158 ,212.962) |
| Dubos (2006)-2 | 0.974 (0.791 ,0.997) | 0.315 (0.243 ,0.397) | 1.421 (1.240 ,1.628) | 0.084 (0.005 ,1.303) | 17.000 (1.001 ,288.765) |
| Dubos (2008) | 0.984 (0.932 ,0.996) | 0.827 (0.741 ,0.888) | 5.676 (3.704 ,8.698) | 0.020 (0.004 ,0.098) | 284.695 (52.326 ,1548.982) |
| Onal(2008) | 0.912 (0.692 ,0.979) | 0.967 (0.747 ,0.997) | 27.353 (1.785 ,419.076) | 0.091 (0.020 ,0.422) | 299.667 (11.279 ,7961.754) |
| Ibrahim (2011) | 0.921 (0.719 ,0.982) | 0.929 (0.741 ,0.983) | 12.895 (2.743 ,60.614) | 0.085 (0.018 ,0.397) | 151.667 (14.415 ,1595.715) |
| Alkholi-1 (2011) | 0.976 (0.808 ,0.998) | 0.643 (0.431 ,0.810) | 2.733 (1.534 ,4.871) | 0.037 (0.002 ,0.583) | 73.800 (3.886 ,1401.565) |
| Alkholi-2 (2011) | 0.881 (0.682 ,0.962) | 0.833 (0.626 ,0.937) | 5.286 (2.005 ,13.932) | 0.143 (0.044 ,0.464) | 37.000 (6.432 ,212.847) |
| Monsef (2012) | 0.722 (0.402 ,0.910) | 0.985 (0.870 ,0.998) | 47.667 (2.956 ,768.654) | 0.282 (0.098 ,0.809) | 169.000 (7.235 ,3947.673) |
| Mayah (2013) | 0.860 (0.677 ,0.947) | 0.878 (0.752 ,0.945) | 7.036 (3.165 ,15.641) | 0.159 (0.060 ,0.424) | 44.117 (10.459 ,186.096) |
| Umran (2014) | 0.783 (0.609 ,0.894) | 0.794 (0.558 ,0.922) | 3.805 (1.468 ,9.861) | 0.273 (0.133 ,0.562) | 13.945 (3.233 ,60.149) |
| Sanaei Dashti (2017) | 0.654 (0.389 ,0.849) | 0.589 (0.407 ,0.750) | 1.592 (0.879 ,2.885) | 0.587 (0.262 ,1.319) | 2.710 (0.690 ,10.648) |
| El Shorbagy-1 (2018) | 0.980 (0.834 ,0.998) | 0.631 (0.480 ,0.760) | 2.655 (1.781 ,3.959) | 0.032 (0.002 ,0.498) | 83.774 (4.754 ,1476.397) |
| El Shorbagy-2 (2018) | 0.860 (0.677 ,0.947) | 0.821 (0.680 ,0.909) | 4.816 (2.470 ,9.389) | 0.170 (0.064 ,0.455) | 28.257 (7.121 ,112.131) |
| Chaudhary (2018) | 0.935 (0.760 ,0.985) | 0.833 (0.654 ,0.930) | 5.609 (2.396 ,13.126) | 0.078 (0.017 ,0.371) | 71.667 (10.298 ,498.766) |
| Garcia-1 (2018) | 0.812 (0.467 ,0.955) | 0.870 (0.811 ,0.913) | 6.273 (3.744 ,10.511) | 0.215 (0.051 ,0.912) | 29.124 (4.661 ,181.969) |
| Garcia-2 (2018) | 0.812 (0.467 ,0.955) | 0.985 (0.953 ,0.995) | 53.950 (15.083 ,192.967) | 0.190 (0.045 ,0.806) | 283.400 (32.335 ,2483.897) |
| Zhang (2019) | 0.250 (0.130 ,0.427) | 0.921 (0.719 ,0.982) | 3.167 (0.604 ,16.591) | 0.814 (0.637 ,1.040) | 3.889 (0.605 ,25.007) |
| **Bivariate summary estimates** | **0.868 (0.777 ,0.925)** | **0.845 (0.754 ,0.907)** | **5.600 (3.159 ,9.946)** | **0.156 (0.296 ,0.083)** | **35.848 (10.680 ,120.283)** |

LR, likelihood ratio; DOR, diagnotic odds ratio.

**Table S3.** Summary estimates of the diagnostic accuracy of blood procalcitonin in studies with a cut-off value ≤ 0.5 ng/mL.

| Study | Sensitivity | Specificity | +LR | -LR | DOR |
|---|---|---|---|---|---|
| Dubos (2006)-1 | 0.868 (0.655, 0.958) | 0.885 (0.82, 0.929) | 7.564 (4.588, 12.471) | 0.149 (0.047, 0.473) | 50.884 (12.158, 212.962) |
| Dubos (2006)-2 | 0.974 (0.791, 0.997) | 0.315 (0.243, 0.397) | 1.421 (1.24, 1.628) | 0.084 (0.005, 1.303) | 17 (1.001, 288.765) |
| Dubos (2008) | 0.984 (0.932, 0.996) | 0.827 (0.741, 0.888) | 5.676 (3.704, 8.698) | 0.02 (0.004, 0.098) | 284.695 (52.326, 1548.982) |
| Onal(2008) | 0.912 (0.692, 0.979) | 0.967 (0.747, 0.997) | 27.353 (1.785, 419.076) | 0.091 (0.02, 0.422) | 299.667 (11.279, 7961.754) |
| Ibrahim (2011) | 0.921 (0.719, 0.982) | 0.929 (0.741, 0.983) | 12.895 (2.743, 60.614) | 0.085 (0.018, 0.397) | 151.667 (14.415, 1595.715) |
| Monsef (2012) | 0.722 (0.402, 0.91) | 0.985 (0.87, 0.998) | 47.667 (2.956, 768.654) | 0.282 (0.098, 0.809) | 169 (7.235, 3947.673) |
| Umran (2014) | 0.783 (0.609, 0.894) | 0.794 (0.558, 0.922) | 3.805 (1.468, 9.861) | 0.273 (0.133, 0.562) | 13.945 (3.233, 60.149) |
| Chaudhary (2018) | 0.935 (0.76, 0.985) | 0.833 (0.654, 0.93) | 5.609 (2.396, 13.126) | 0.078 (0.017, 0.371) | 71.667 (10.298, 498.766) |
| Garcia-1 (2018) | 0.812 (0.467, 0.955) | 0.87 (0.811, 0.913) | 6.273 (3.744, 10.511) | 0.215 (0.051, 0.912) | 29.124 (4.661, 181.969) |
| Bivariate summary estimates | 0.899 (0.81, 0.949) | 0.844 (0.702, 0.96) | 5.763 (2.718, 23.725) | 0.12 (0.271, 0.053) | 48.157 (10.043, 446.588) |

LR, likelihood ratio; DOR, diagnotic odds ratio.

**Table S4.** Heterogeneity among studies with a cut-off value ≤ 0.5 ng/mL.

| Sensitivity | Specificity |
|---|---|
| $\chi^2$ = 19.4679, p-value=0.0125 | $\chi^2$ = 187.4487 p-value<0.0001 |

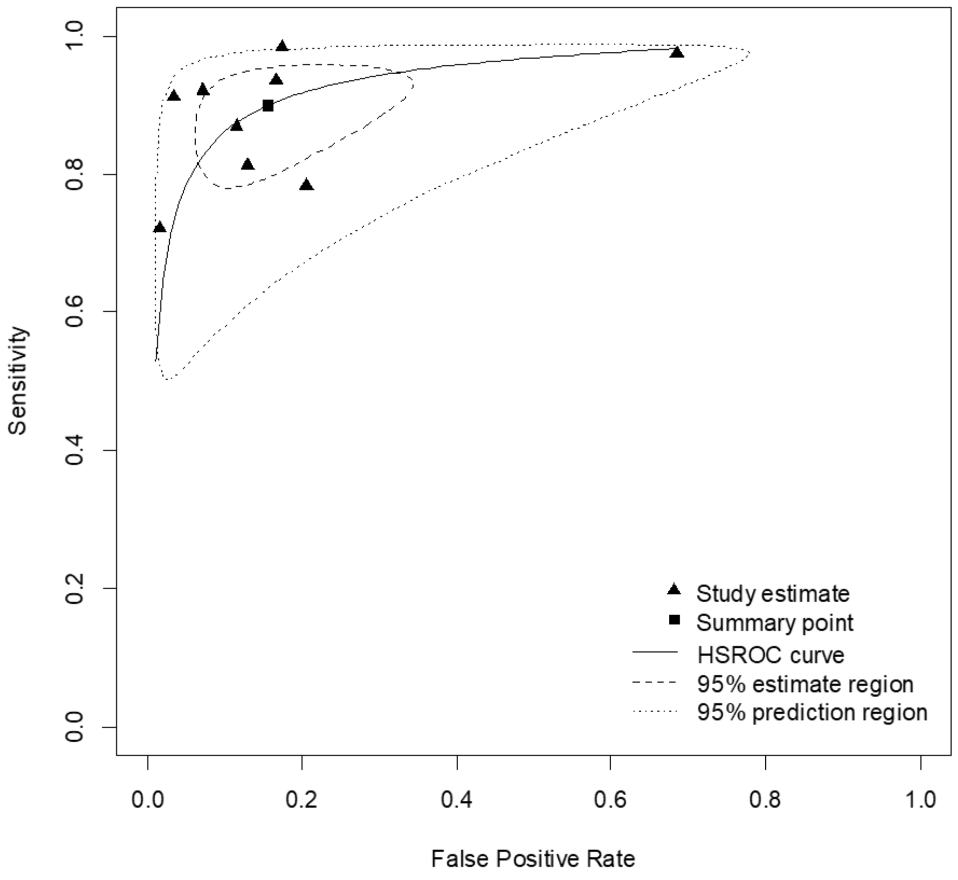


**Figure S1.** Hierarchical summary receiver operating characteristic (HSROC) curve in studies with a cut-off value ≤ 0.5 ng/mL. The area under the HSROC curve was 0.935.

**Table S5.** Summary estimates of the diagnostic accuracy of blood procalcitonin in studies with a cut-off value > 0.5 ng/mL.

| Study | Sensitivity | Specificity | +LR | -LR | DOR |
|---|---|---|---|---|---|
| Gendrel (1997) | 0.921 (0.719, 0.982) | 0.988 (0.896, 0.999) | 77.368 (4.905, 1220.458) | 0.08 (0.017, 0.371) | 968.333 (37.584, 24948.556) |
| Alkholi-1 (2011) | 0.976 (0.808, 0.998) | 0.643 (0.431, 0.81) | 2.733 (1.534, 4.871) | 0.037 (0.002, 0.583) | 73.8 (3.886, 1401.565) |
| Alkholi-2 (2011) | 0.881 (0.682, 0.962) | 0.833 (0.626, 0.937) | 5.286 (2.005, 13.932) | 0.143 (0.044, 0.464) | 37 (6.432, 212.847) |
| Mayah (2013) | 0.86 (0.677, 0.947) | 0.878 (0.752, 0.945) | 7.036 (3.165, 15.641) | 0.159 (0.06, 0.424) | 44.117 (10.459, 186.096) |
| Sanaei Dashti (2017) | 0.654 (0.389, 0.849) | 0.589 (0.407, 0.75) | 1.592 (0.879, 2.885) | 0.587 (0.262, 1.319) | 2.71 (0.69, 10.648) |
| El Shorbagy-1 (2018) | 0.98 (0.834, 0.998) | 0.631 (0.48, 0.76) | 2.655 (1.781, 3.959) | 0.032 (0.002, 0.498) | 83.774 (4.754, 1476.397) |
| El Shorbagy-2 (2018) | 0.86 (0.677, 0.947) | 0.821 (0.68, 0.909) | 4.816 (2.47, 9.389) | 0.17 (0.064, 0.455) | 28.257 (7.121, 112.131) |
| Garcia-2 (2018) | 0.812 (0.467, 0.955) | 0.985 (0.953, 0.995) | 53.95 (15.083, 192.967) | 0.19 (0.045, 0.806) | 283.4 (32.335, 2483.897) |
| Zhang (2019) | 0.25 (0.13, 0.427) | 0.921 (0.719, 0.982) | 3.167 (0.604, 16.591) | 0.814 (0.637, 1.04) | 3.889 (0.605, 25.007) |
| Bivariate summary estimates | 0.831 (0.647, 0.93) | 0.851 (0.706, 0.931) | 5.577 (2.201, 13.478) | 0.199 (0.5, 0.075) | 28.084 (4.401, 179.261) |

LR, likelihood ratio; DOR, diagnotic odds ratio

**Table S6.** Heterogeneity among studies with a cut-off value > 0.5 ng/mL.

| Sensitivity | Specificity |
|---|---|
| $\chi^2$ = 66.9558, p-value<0.001 | $\chi^2$ = 76.3249 p-value<0.0001 |

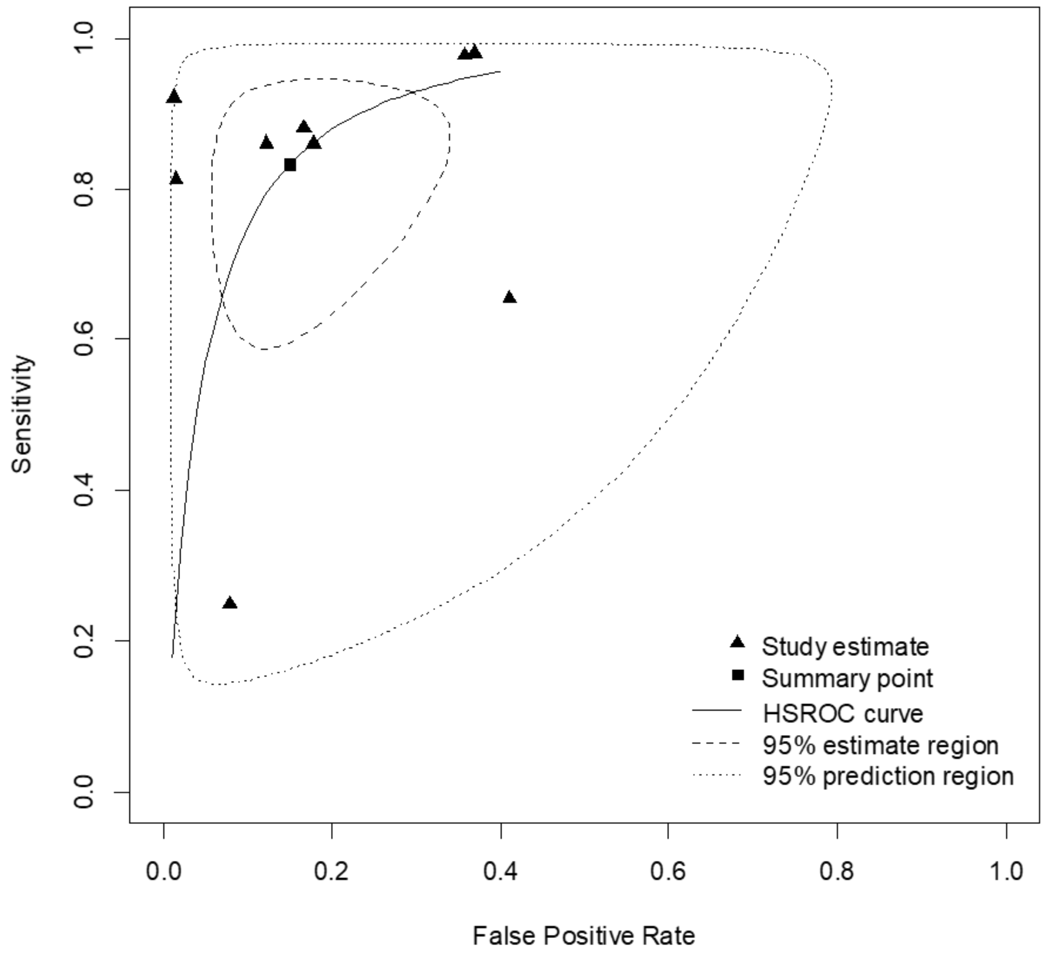


**Figure S2.** Hierarchical summary receiver operating characteristic (HSROC) curve in studies with a cut-off value > 0.5 ng/mL. The area under the HSROC curve was 0.908.

**Table S7.** Diagnostic performance of C-reactive protein.

| Study | Sensitivity | Specificity | +LR | -LR | DOR |
|---|---|---|---|---|---|
| Dubos (2006) | 0.886 (0.694, 0.964) | 0.711 (0.633, 0.778) | 3.066 (2.284, 4.115) | 0.16 (0.05, 0.516) | 19.179 (4.902, 75.03) |
| Dubos (2008) | 0.828 (0.74, 0.891) | 0.665 (0.569, 0.749) | 2.472 (1.856, 3.294) | 0.258 (0.163, 0.409) | 9.567 (4.897, 18.691) |
| Ibrahim (2011) | 0.763 (0.539, 0.899) | 0.881 (0.682, 0.962) | 6.411 (1.95, 21.075) | 0.269 (0.118, 0.612) | 23.844 (4.391, 129.481) |
| Alkholi-1 (2011) | 0.881 (0.682, 0.962) | 0.595 (0.387, 0.774) | 2.176 (1.266, 3.742) | 0.2 (0.059, 0.675) | 10.882 (2.236, 52.952) |
| Alkholi-2 (2011) | 0.738 (0.525, 0.878) | 0.786 (0.574, 0.909) | 3.444 (1.461, 8.121) | 0.333 (0.157, 0.707) | 10.333 (2.483, 42.997) |
| Mayah (2013) | 0.804 (0.605, 0.917) | 0.793 (0.656, 0.885) | 3.895 (2.135, 7.105) | 0.247 (0.106, 0.572) | 15.795 (4.51, 55.317) |
| Umran (2014) | 0.75 (0.573, 0.87) | 0.735 (0.498, 0.886) | 2.833 (1.249, 6.425) | 0.34 (0.172, 0.673) | 8.333 (2.143, 32.4) |
| Sanaei Dashti (2017) | 0.708 (0.428, 0.887) | 0.983 (0.854, 0.998) | 41.083 (2.57, 656.743) | 0.297 (0.123, 0.718) | 138.429 (6.487, 2954.081) |
| El Shorbagy-1 (2018) | 0.86 (0.677, 0.947) | 0.607 (0.457, 0.74) | 2.189 (1.456, 3.292) | 0.231 (0.085, 0.628) | 9.494 (2.618, 34.429) |
| El Shorbagy-2 (2018) | 0.74 (0.545, 0.871) | 0.774 (0.628, 0.874) | 3.272 (1.785, 5.995) | 0.336 (0.17, 0.664) | 9.737 (3.085, 30.733) |
| Bivariate Summary estimates | 0.797 (0.741, 0.844) | 0.725 (0.665, 0.777) | 2.894 (2.213, 3.781) | 0.28 (0.39, 0.201) | 10.334 (5.679, 18.808) |

LR, likelihood ratio; DOR, diagnotic odds ratio.

**Table S8.** Heterogeneity among studies evaluating C-reactive protein level.

| Sensitivity | | Specificity | |
|---|---|---|---|
| $\chi^2$=5.305 | p-value=0.807 | $\chi^2$=21.032 | p-value=0.013 |

**Table S9.** Diagnostic performance of white blood cell count.

| Study | Sensitivity | Specificity | +LR | -LR | DOR |
|---|---|---|---|---|---|
| Dubos (2006) | 0.619 (0.409, 0.792) | 0.819 (0.749, 0.874) | 3.429 (2.114, 5.56) | 0.465 (0.268, 0.806) | 7.375 (2.774, 19.606) |
| Dubos (2008) | 0.479 (0.382, 0.578) | 0.784 (0.695, 0.853) | 2.222 (1.453, 3.397) | 0.664 (0.534, 0.825) | 3.345 (1.802, 6.212) |
| Ibrahim (2011) | 0.722 (0.491, 0.875) | 0.65 (0.433, 0.819) | 2.063 (1.064, 4.002) | 0.427 (0.19, 0.962) | 4.829 (1.213, 19.219) |
| Umran (2014) | 0.724 (0.543, 0.853) | 0.75 (0.505, 0.898) | 2.897 (1.204, 6.969) | 0.368 (0.191, 0.707) | 7.875 (1.953, 31.748) |
| Chaudhary (2018) | 0.818 (0.615, 0.927) | 0.5 (0.326, 0.674) | 1.636 (1.076, 2.489) | 0.364 (0.139, 0.95) | 4.5 (1.211, 16.719) |
| Bivariate Summary estimates | 0.659 (0.504, 0.786) | 0.713 (0.587, 0.813) | 2.294 (1.22, 4.195) | 0.479 (0.845, 0.263) | 4.794 (1.443, 15.925) |

LR, likelihood ratio; DOR, diagnotic odds ratio.

**Table S10.** Heterogeneity among studies evaluating white blood cell count.

| Sensitivity | | Specificity | |
|---|---|---|---|
| $\chi^2$=13.175 | p-value=0.0105 | $\chi^2$=14.993 | p-value=0.0047 |

**Table S11.** Diagnostic performance of white blood cell count in the cerebrospinal fluid.

| Study | Sensitivity | Specificity | +LR | -LR | DOR |
|---|---|---|---|---|---|
| Dubos (2006) | 0.762 (0.549, 0.894) | 0.747 (0.67, 0.81) | 3.006 (2.083, 4.339) | 0.319 (0.148, 0.689) | 9.427 (3.23, 27.516) |
| Dubos (2008) | 0.792 (0.7, 0.861) | 0.686 (0.591, 0.768) | 2.523 (1.86, 3.423) | 0.304 (0.201, 0.458) | 8.312 (4.356, 15.862) |
| Sanaei Dashti (2017) | 0.833 (0.552, 0.953) | 0.553 (0.397, 0.699) | 1.863 (1.206, 2.877) | 0.302 (0.082, 1.103) | 6.176 (1.189, 32.076) |
| Chaudhary (2018) | 0.545 (0.347, 0.731) | 0.625 (0.427, 0.788) | 1.455 (0.765, 2.764) | 0.727 (0.418, 1.264) | 2.00 (0.616, 6.494) |
| Bivariate Summary estimates | 0.733 (0.601, 0.834) | 0.669 (0.58, 0.748) | 2.217 (1.43, 3.309) | 0.399 (0.689, 0.223) | 5.556 (2.076, 14.87) |

LR, likelihood ratio; DOR, diagnotic odds ratio.

**Table S12.** Heterogeneity among studies evaluating white blood cell count in the cerebrospinal fluid.

| Sensitivity | | Specificity | |
|---|---|---|---|
| $\chi^2$=6.323 | p-value=0.0969 | $\chi^2$=6.037 | p-value=0.11 |

**Table S13.** Diagnostic performance of neutrophils in the cerebrospinal fluid.

| Study | Sensitivity | Specificity | +LR | -LR | DOR |
|---|---|---|---|---|---|
| Dubos (2006) | 0.795 (0.59, 0.913) | 0.809 (0.737, 0.865) | 4.165 (2.799, 6.198) | 0.253 (0.11, 0.579) | 16.475 (5.396, 50.3) |
| Dubos (2008) | 0.818 (0.729, 0.882) | 0.728 (0.628, 0.809) | 3.004 (2.115, 4.266) | 0.25 (0.161, 0.39) | 11.992 (5.981, 24.044) |
| Sanaei Dashti (2017) | 0.962 (0.717, 0.996) | 0.423 (0.282, 0.578) | 1.667 (1.247, 2.227) | 0.091 (0.006, 1.412) | 18.333 (1.012, 332.262) |
| Chaudhary (2018) | 0.283 (0.141, 0.487) | 0.865 (0.687, 0.95) | 2.099 (0.65, 6.778) | 0.829 (0.615, 1.117) | 2.532 (0.596, 10.758) |
| Bivariate Summary estimates | 0.793 (0.377, 0.96) | 0.749 (0.519, 0.892) | 3.158 (0.784, 8.895) | 0.277 (1.201, 0.045) | 11.403 (0.652, 199.271) |

LR, likelihood ratio; DOR, diagnotic odds ratio.

**Table S14.** Heterogeneity among studies evaluating neutrophils in the cerebrospinal fluid.

| Sensitivity | | Specificity | |
|---|---|---|---|
| $\chi^2$=32.203 | p-value<0.0001 | $\chi^2$=26.062 | p-value<0.0001 |

**Table S15.** Diagnostic performance of protein level in the cerebrospinal fluid.

| Study | Sensitivity | Specificity | +LR | -LR | DOR |
|---|---|---|---|---|---|
| Dubos-1 (2006) | 0.841 (0.64, 0.94) | 0.781 (0.707, 0.841) | 3.844 (2.687, 5.5) | 0.204 (0.078, 0.534) | 18.878 (5.636, 63.232) |
| Dubos-2 (2006) | 0.977 (0.815, 0.998) | 0.587 (0.505, 0.664) | 2.365 (1.927, 2.903) | 0.039 (0.002, 0.602) | 61.067 (3.628, 1028.01) |
| Dubos (2008) | 0.88 (0.8, 0.931) | 0.649 (0.552, 0.735) | 2.504 (1.902, 3.297) | 0.185 (0.105, 0.324) | 13.557 (6.474, 28.391) |
| Chaudhary (2018) | 0.674 (0.47, 0.828) | 0.574 (0.39, 0.74) | 1.582 (0.939, 2.667) | 0.568 (0.29, 1.112) | 2.786 (0.875, 8.871) |
| Bivariate Summary estimates | 0.838 (0.699, 0.92) | 0.658 (0.55, 0.753) | 2.452 (1.552, 3.718) | 0.247 (0.548, 0.107) | 9.934 (2.833, 34.833) |

LR, likelihood ratio; DOR, diagnotic odds ratio.

**Table S16.** Heterogeneity among studies evaluating protein level in the cerebrospinal fluid.

| Sensitivity | | Specificity | |
|---|---|---|---|
| $\chi^2$=9.456 | p-value=0.0238 | $\chi^2$=13.860 | p-value=0.0031 |

**Table S17.** Diagnostic performance of glucose level in the cerebrospinal fluid.

| Study | Sensitivity | Specificity | +LR | -LR | DOR |
|---|---|---|---|---|---|
| Dubos (2006) | 0.381 (0.208, 0.591) | 0.224 (0.163, 0.299) | 0.491 (0.283, 0.853) | 2.766 (1.758, 4.354) | 0.177 (0.068, 0.465) |
| Dubos (2008) | 0.326 (0.24, 0.426) | 0.182 (0.118, 0.269) | 0.399 (0.294, 0.54) | 3.705 (2.385, 5.757) | 0.108 (0.055, 0.21) |
| Chaudhary (2018) | 0.909 (0.722, 0.975) | 0.154 (0.062, 0.335) | 1.074 (0.87, 1.326) | 0.591 (0.119, 2.926) | 1.818 (0.3, 11.023) |
| Bivariate Summary estimates | 0.563 (0.172, 0.889) | 0.193 (0.145, 0.253) | 0.698 (0.201, 1.189) | 2.264 (5.718, 0.44) | 0.308 (0.035, 2.701) |

LR, likelihood ratio; DOR, diagnotic odds ratio.

**Table S18.** Heterogeneity among studies evaluating glucose level in the cerebrospinal fluid.

| Sensitivity | | Specificity | |
|---|---|---|---|
| $\chi^2$=25.007 | p-value<0.0001 | $\chi^2$=1.0464 | p-value=0.593 |
